# A Rare Case of Klinefelter Syndrome Patient with Quintuple Mosaic Karyotype, Diagnosed by GTG-Banding and FISH

**Published:** 2014-07-08

**Authors:** Hamideh Karimi, Marjan Sabbaghian, Kaveh Haratian, Hamed Vaziri Nasab, Faramarz Farrahi, Shabnam Zari Moradi, Tayebeh Tavakolzadeh, Zahra Beheshti, Hamid Gourabi, Anahita Mohseni Meybodi

**Affiliations:** 1 Department of Genetics at Reproductive Biomedicine Research Center, Royan Institute for Reproductive Biomedicine, ACECR, Tehran, Iran; 2 Department of Andrology at Reproductive Biomedicine Research Center, Royan Institute for Reproductive Biomedicine, ACECR, Tehran, Iran; 3 Department of Pathobiology, Faculty of Medicine, Alborz University of Medical Science, Karaj, Iran

**Keywords:** Klinefelter Syndrome, Karyotype, Mosaicism, Fluorescent in situ Hybridization

## Abstract

Klinefelter syndrome (KS) is the most common sex chromosomal disorder in men. Most of these patients show the 47,XXY karyotype, whereas approximately 15% of them are mosaics with variable phenotype. A 39-year-old male investigated for primary infertility, was clinically normal with small firm testes and elevated levels of FSH, LH and low level of testosterone. Total azoospermia was confirmed on semen analysis. Testicular histopathology revealed no spermatogenesis and absence of germ cells. Karyotype from whole blood culture showed cells with 47,XXY/46,XX/ 45,X/48,XXXY/ 46,XY mosaicism. The predominant cell line was 47,XXY (83.67%). This was confirmed by fluorescence in situ hybridization (FISH). Also the presence of a small population of cells with the 48,XXXY and 45,X karyotypes was detected by FISH. This case illustrates the utility of FISH as an adjunct to conventional cytogenetics in assess the chromosome copy number in each cell line of a mosaic.

## Introduction

Klinefelter syndrome (KS), a chromosomal disorder due to an extra X chromosome (47,XXY) (1), represents the most commonly found human sex chromosomal abnormality with an incidence of one in 500 newborn males. It is characterized by hypogonadism, gynecomastia, azoospermia or oligospermia, and increased levels of gonadotropins (2). KS is the most frequent genetic cause of male infertility (4-6%) and it is observed in up to 11% of azoospermic and 0.7% of oligospermic men (3).

Small number of 47,XXY patients have sperm production that would allow them to benefit from assisted reproductive techniques (ART) such as micro dissection testicular sperm extraction (TESE) and intracytoplasmic sperm injection (ICSI) (4). Although most KS patients have a non-mosaic 47,XXY karyotype in all body cells, a mosaic 47,XXY/46, XY karyotype is found in about 10-15% of cases (1, 5). Rarely, multiple line mosaics can be found (5, 6). The clinical features are variable and when a mosaic for a 46,XY cell line is present, it is associated with a broad spectrum of fertility-associated problems, ranging from azoospermia to different grades of testicular insufficiency. This variation most likely depends on the number of abnormal cells and their location in body tissues (5). In most cases with no evidence of mosaicism, no sperm in the ejaculate can be found (6).

We report on an interesting case of a KS patient with a new type of quintuple mosaicism in peripheral blood lymphocytes.

## Case Report

A 39 year old man was referred to Royan Institute cytogenetic laboratory suffering from infertility. He was born from a full term natural delivery with no apparent complication. The age of his mother at this pregnancy was 35 and his father was 40. The parents were unrelated. Family history of infertility was negative and his only brother fathered a child. On examination he was 180 cm, 82 kg. Each testis volume was 4 ml estimated by Prader’s orchidometer (normal range: 15-25 ml), with normal vas deferens. He had a history of right sided epididymo-orchitis. Stature growth was regular and puberty was normal without testosterone therapy. Motor and mental development of the patient was normal. There were no malformations, no gynecomastia, no diabetes and no reduced muscle strength. Olfaction was normal. The semen analysis showed total azoospermia with low volume (0.3 ml) and normal pH and fructose level. No spermatozoa were found in micro dissection TESE (MD-TESE) and the seminiferous tubules were hyalinized. Histology of testis biopsy specimen showed only Sertoli cells and moderate hyperplasia of the leydig cells. Endocrinological laboratory studies revealed elevated follicle stimulating hormone (FSH=43 mIU/ml, reference 0.9-8.9 mIU/ml) and luteinizing hormone (LH=14.3 mIU/ml, reference 0.8-10 mIU/ml) levels and low testosterone levels (1.4 ng/ml). The results were suggestive for hypergonadotropic hypogonadism and KS was the most probable diagnosis.

### Cytogenetic analysis

Chromosomal analysis was performed on phytohemagglutinin-stimulated peripheral lymphocyte cultures using standard cytogenetic methods. Two different cultures for the sample prepared and two different series of slides from each culture analyzed separately. Half of slides were investigated by GTG and the other half by FISH. 170 GTG banded metaphases from the patient were analyzed at the resolution of 550 bands.

**Fig 1:**
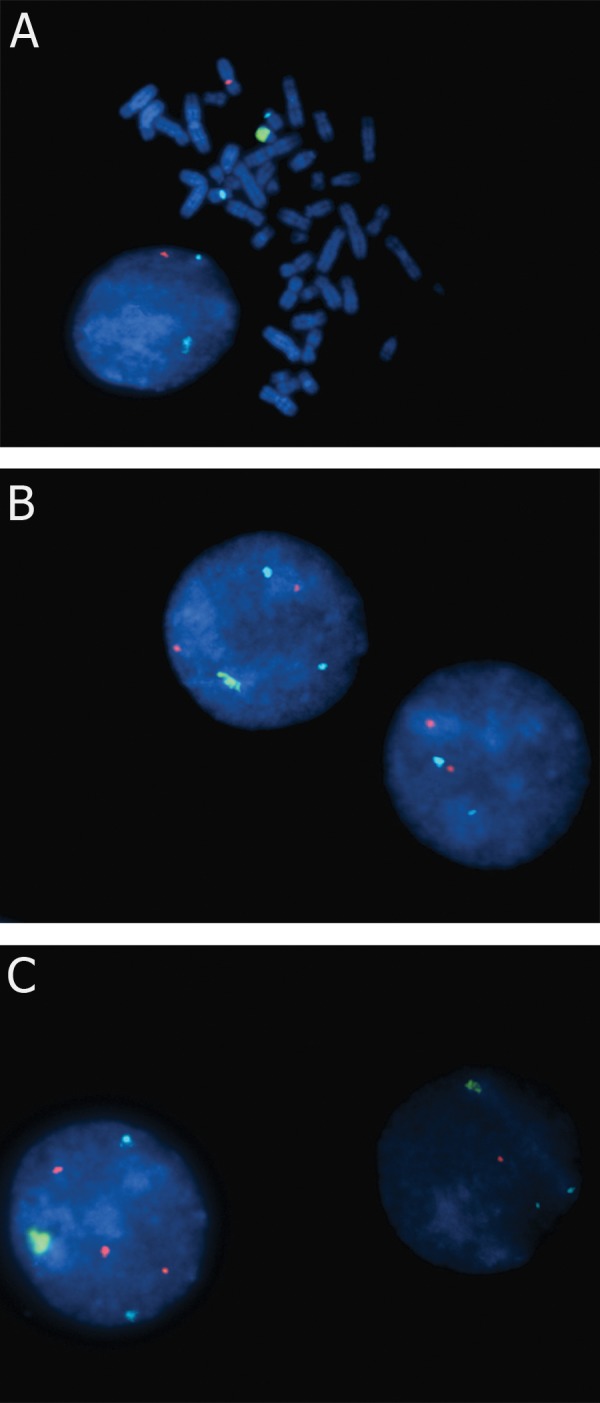
FISH of interphase cells and mitotic lymphocytes with different probes: Yq Telomere probe (G); X Centromere probe (O) ; 18 Centromere probe (A) A. 46,XY metaphase and a 45,X interphase cell. B. 46,XX and a 47,XXY interphase cell. C. 48,XXXY and a 46,XY interphase cell.

The hybridization on metaphase chromosomes was performed according to standard cytogenetic protocols (7). A triple-colour FISH with centromeric DNA probes for chromosomes X (CEP X SpectrumOrange) and telomeric DNA Probes for chromosomes Y telomer (Yq1.2-Satellite III SpectrumGreen, Direct Labeled Fluorescent DNA Probe Kit, Vysis, Abbott Molecular, USA) was used to determine the sex chromosome constitution of metaphase lymphocytes. Centromeric DNA probes for chromosomes 18 (CEP 18 SpectrumAqua) were used as control for binding efficiency (Fig 1). 50 metaphase nuclei and 80 cells in interphase were scored by FISH and subsequent results are briefly given in table 1.

Accordingly, the karyotype of the case was ascertained as: 47,XXY[251]/46,XX[10]/45, X[10]/48,XXXY[7]/46,XY[22] according to The International System for Human Cytogenetic Nomenclature ISCN 2009 (8). Molecular analysis showed no microdeletions in the Y chromosome.

**Table 1:** Number of each cell line analyzed by GTG banding and FISH


Total	FISH Results		GTG Results	Cell line
	Metaphase	Interphase		

10 (3.33%)	2	5	3	X,45
7 (2.34%)	2	4	1	XXXY,48
10 (3.33%)	3	3	4	XX,46
251 (83.67%)	39	62	150	XXY,47
22 (7.33%)	4	6	12	XY,46
300	50	80	170	Total


GTG banding; G-band by Trypsin using Giemsa and FISH; Fluorescence in situ hybridization.

## Discussion

Mosaicism involving more than three lines is rarely detected and the predominant cell line differs between different cases. The origin of the quintuple mosaicism may be explained by meiotic and mitotic disturbances in the formation of the zygote and embryo. Regarding the possible explanations for mosaicism, the most reasonable hypothesis is a nondisjunctional event. The predominance of the 47,XXY and the presence of 45,X cell lines in our patient strongly suggest the mitotic nondisjunction of the X chromosome in a normal XY zygote results in a 47,XXY and a 45,X clone, with successive nondisjunction of the 47,XXY cell line producing 48,XXXY cells. The XX cell line could be explained by the loss of Y chromosome in a proportion of the XXY cells. It is well known that both the phenotypic sex and gonadal phenotype are influenced by the percentage and distribution of Y-carrying cells in the gonad, but not necessarily in the blood (9). If this presence of cells with 46,XX karyotype has any connection with the MD-TESE and testis histology results of the patient, it cannot be determined because unfortunately the karyotype of other tissues specially the gonads were not available for this patient.

The only quintuple mosaicism in patients with Klinefelter’s syndrome (with the cell lines different from ours) has been reported before by Al-Awadi et al. (10). Our patient shows a rare chromosome variant causing KS with a relatively normal phenotype. The final cytogenetic diagnosis of this patient is established as 47,XXY/46,XX/45,X/48,XXXY/ 46,XY. To our knowledge this is the first quintuple mosaic case with this pattern.

A man with KS may wish to reproduce with the aid of modern reproductive technologies. TESE-ICSI procedure has offered a new hope to those patients (11, 12). The first child using ICSI for a non-mosaic Klinefelter man was born in 1997(13). Since then many more births have been reported (14, 15). Some studies have shown that there is direct correlation between the rate of gonosomal mosaicism in somatic cells and fertility in 47, XXY patients with an increased incidence of XY cells in their lymphocytes (16). Therefore obtaining a sharp and clear karyotype might help to estimate the frequency of abnormal germ cells for a risk estimation and genetic counseling. In this case, 45,X cell karyotype was firstly detected by FISH whereas it had not been detected in the initial 50 GTG-banded analyzed cells. Moreover, FISH confirmed the presence of 48,XXXY cell population represented in a low percentage, which was just seen once in the original karyotype study, probably due to its low presence in peripheral blood and a difficulty for mitotic divisions (Table 1). These findings also show the utility of FISH as an important tool that helps conventional cytogenetics to establish the number of chromosomes in each cell line of a mosaic and detects low-percentage mosaicisms. FISH was also recommended for Klinefelter’s syndrome patients to define exactly the cytogenetic status as mosaic or non-mosaic by Abdelmoula et al. (17).

Unfortunately in our patient, there was no sign of spermatogenesis, concluding that he could not profit from assisted reproduction techniques.

## Conclusion

Through a combination of GTG-banding and FISH, the karyotype of the patient was determined. FISH is recommended in mosaic forms of KS to exactly define the cytogenetic status of the patient. Generally most individuals with KS have germ cells with sex chromosomal abnormalities and may wish to reproduce with ART, thus it is important that accurate estimation of the frequency of abnormal cells be obtained for fertility counseling, prognosis discussion and risk estimation.

## References

[1] Fruhmesser A, Kotzot D (2011). Chromosomal variants in klinefelter syndrome. Sex Dev.

[2] Schinzel A (2001). Catalogue of unbalanced chromosome aberrations in man.

[3] De Braekeleer M, Dao TN (1991). Cytogenetic studies in male infertility: a review. Hum Reprod.

[4] Denschlag D, Tempfer C, Kunze M, Wolff G, Keck C (2004). Assisted reproductive techniques in patients with Klinefelter syndrome: a critical review. Fertil steril.

[5] Mark HF, Bai H, Sotomayor E, Mark S, Zolnierz K, Airall E (1999). A variant Klinefelter syndrome patient with an XXY/XX/XY karyotype studied by GTG-banding and fluorescence in situ hybridization. Exp Mol Pathol.

[6] Zamora L, Espinet B, Salido M, Sole F, Ligorria C, Florensa L (2002). Report of 46,XX/46,XY/47,XXY/48,XXYY mosaicism in an adult phenotypic male. Am J Med Genet.

[7] Amiel A, Fejgin M, Appelman Z, Shapiro I, Gaber E, Bachar A (1995). Fluorescent in-situ hybridization (FISH) as an aid to marker chromosome identification in prenatal diagnosis. Eur J Obstet Gynecol Reprod Biol.

[8] Shaffer LG TN (2009). An International System for Human Cytogenetic Nomenclature (ISCN).

[9] Velissariou V, Christopoulou S, Karadimas C, Pihos I, Kanaka-Gantenbein C, Kapranos N (2006). Rare XXY/XX mosaicism in a phenotypic male with Klinefelter syndrome: case report. Eur J Med Genet.

[10] Al-Awadi SA, Teebi AS, Krishna Murthy DS, Othman G, Sundareshan TS (1986). Klinefelter’s syndrome, mosaic 46,XX/46,XY/47,XXY/48,XXXY/48,XXYY: a case report. Ann Genet.

[11] Aboulfotouh II, Youssef MA, Mady AF, Abdelhak AM, Khattab SM (2011). Non-mosaic Klinefelter syndrome and successful testicular sperm extraction-intracytoplasmic sperm injection procedure: case report. Gynecol Endocrinol.

[12] Sciurano RB, Luna Hisano CV, Rahn MI, Brugo Olmedo S, Rey Valzacchi G, Coco R (2009). Focal spermatogenesis originates in euploid germ cells in classical Klinefelter patients. Hum Reprod.

[13] Bourne H, Stern K, Clarke G, Pertile M, Speirs A, Baker HW (1997). Delivery of normal twins following the intracytoplasmic injection of spermatozoa from a patient with 47,XXY Klinefelter’s syndrome. Hum Reprod.

[14] Greco E, Iacobelli M, Rienzi L, Fabris GF, Tesorio N, Tesarik J (2008). Birth of a healthy boy after fertilization of cryopreserved oocytes with cryopreserved testicular spermatozoa from a man with nonmosaic Klinefelter syndrome. Fertil Steril.

[15] Kyono K, Uto H, Nakajo Y, Kumagai S, Araki Y, Kanto S (2007). Seven pregnancies and deliveries from non-mosaic Klinefelter syndrome patients using fresh and frozen testicular sperm. J Assist Reprod Genet.

[16] Lenz P, Luetjens CM, Kamischke A, Kuhnert B, Kennerknecht I, Nieschlag E (2005). Mosaic status in lymphocytes of infertile men with or without Klinefelter syndrome. Hum Reprod.

[17] Abdelmoula NB, Amouri A, Portnoi MF, Saad A, Boudawara T, Mhiri MN (2004). Cytogenetics and fluorescence in situ hybridization assessment of sex-chromosome mosaicism in Klinefelter’s syndrome. Ann Genet.

